# Thalamus anatomy predicts cognitive performance and hippocampal atrophy in aging adults: a UK Biobank study

**DOI:** 10.1093/braincomms/fcaf334

**Published:** 2025-09-10

**Authors:** Guocheng Jiang, Fa-Hsuan Lin, Hugo Cogo-Moreira, Jennifer S Rabin, J Jean Chen, Walter Swardfager, Bradley J MacIntosh

**Affiliations:** Department of Medical Biophysics, University of Toronto, Toronto M5S 1A1, Canada; Sandra Black Centre for Brain Resilience and Recovery, Hurvitz Brain Sciences Program, Sunnybrook Research Institute, Toronto, ON M4N 3M5, Canada; Department of Medical Biophysics, University of Toronto, Toronto M5S 1A1, Canada; Physical Sciences Platform, Sunnybrook Research Institute, Toronto M4N 3M5, Canada; Institute of Medical Science, University of Toronto, Toronto, ON M5S 1A1, Canada; Faculty of Teacher Education and Languages, Østfold University College, Halden 1757, Norway; Hurvitz Brain Sciences Program, Sunnybrook Research Institute, Toronto, Ontario M4N 3M5, Canada; Department of Psychology, University of Toronto, Toronto, M5S 1A1, Canada; Department of Medical Biophysics, University of Toronto, Toronto M5S 1A1, Canada; Baycrest Health Sciences, Rotman Research Institute, Toronto M6A 2E1, Canada; Institute of Biomedical Engineering, Faculty of Applied Science and Engineering, University of Toronto, Toronto M5S 3E3, Canada; Sandra Black Centre for Brain Resilience and Recovery, Hurvitz Brain Sciences Program, Sunnybrook Research Institute, Toronto, ON M4N 3M5, Canada; Department of Pharmacology and Toxicology, University of Toronto, Toronto, Ontario M5S 1A1, Canada; KITE Research Institute, University Health Network, Toronto, Ontario M5G 2A2, Canada; Department of Medical Biophysics, University of Toronto, Toronto M5S 1A1, Canada; Sandra Black Centre for Brain Resilience and Recovery, Hurvitz Brain Sciences Program, Sunnybrook Research Institute, Toronto, ON M4N 3M5, Canada; Physical Sciences Platform, Sunnybrook Research Institute, Toronto M4N 3M5, Canada; Hurvitz Brain Sciences Program, Sunnybrook Research Institute, Toronto, Ontario M4N 3M5, Canada; Department of Physics and Computational Radiology, Computational Radiology and Artificial Intelligence Unit, Clinic for Radiology and Nuclear Medicine, Oslo University Hospital, Oslo N-0424, Norway

**Keywords:** thalamus, hippocampus, cognitive decline, healthy brain aging, longitudinal brain MRI

## Abstract

The thalamus has extensive inter-connectedness with different brain regions in serving cognitive processes. In a community-dwelling aging population from the United Kingdom, this study examined the independent contribution of thalamus volume loss to cognitive performances and the longitudinal anatomical relationship between the thalamus and the interconnected hippocampus. We accessed MRI data from 4348 cognitively unimpaired older adults from the UK Biobank, of whom 653 participants had follow-up MRI. We estimated regional brain volumes using T1-weighted MRI. Linear models tested the association between the thalamus volume and a cognitive composite score derived from digit-symbol substitution and trail-making tests. We used latent change score models to test the longitudinal associations between thalamus volume at baseline and the trajectory of hippocampal atrophy, and vice versa. Baseline thalamus volume was positively associated with the cognitive composite score (β=0.055±0.018, *P* = 0.002, *R*^2^ = 0.09). A larger baseline thalamus volume predicted slower hippocampal atrophy ($\gamma_{T\to \mathrm{dH}}=-0.048\pm 0.015,\;P=0.001,\;\;R^{2}=0.09$), while larger hippocampal volume at baseline predicted faster thalamic atrophy ($\gamma_{H\to dT}=0.043\pm 0.022,\;P=0.048,\;R^{2}=0.04$). Sex-stratified analysis revealed that hippocampal volume significantly predicted thalamic atrophy only in women. This study revealed that thalamic volume loss was associated with impaired processing speed and executive function. Thalamus and hippocampus anatomy showed bidirectional longitudinal associations and demonstrated sex differences. These findings underscore the thalamus anatomy as an important marker of brain health in the aging population.

## Teaser

Using UK Biobank brain MRI data from community-dwelling older adults, Jiang *et al*. reported that thalamus volume was associated with processing speed and executive function. Longitudinal analyses revealed bidirectional, sex-specific links between hippocampal and thalamic anatomy, highlighting the thalamus as a key hub region in healthy brain aging.

## Graphical Abstract

**Graphical Abstract:**
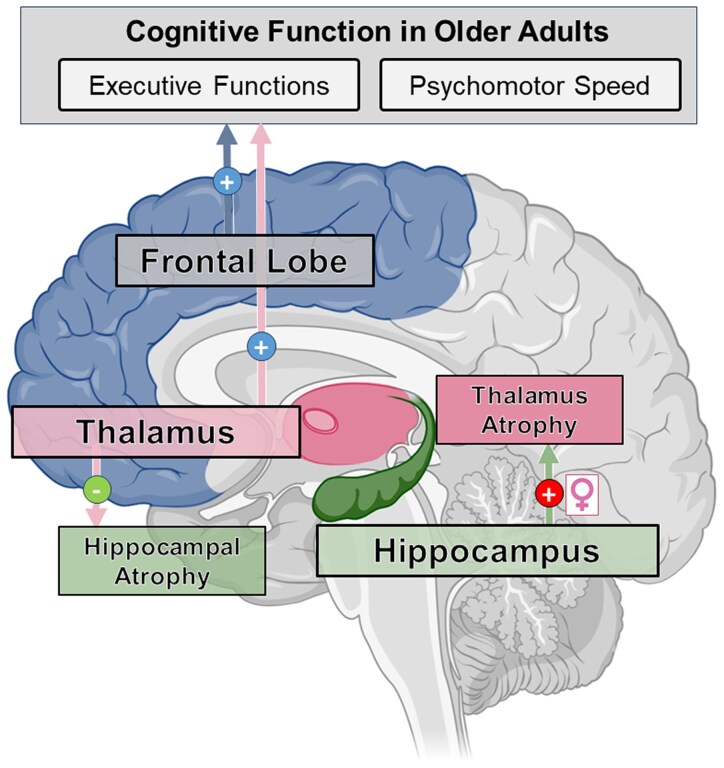

## Introduction

The thalamus is a grey matter (GM) subcortical structure containing multiple nuclei, each projecting to distinct brain regions.^1^ It is a central relay station that projects and sorts neuronal activation to higher cortical and subcortical regions.^1^ Age-related brain volume loss is associated with cognitive decline in older adults. Classically, impaired executive functioning and psychomotor speed have been associated with volume loss in the cerebral cortex, including the prefrontal cortex and parietal regions.^2^ Recent studies suggest that subcortical structures, including the thalamus and basal ganglia, may also contribute to the performance of cognitive processes.^3^ The mediodorsal nuclei of the thalamus showed a reciprocal connection to the prefrontal cortex.^1^ Volume loss on either node of the circuit may contribute to impaired executive function and processing speed.^1^ Literature supports the notion that the thalamus contributes to impaired cognition in attention, memory, and processing speed in healthy older adults and patients with neurodegenerative diseases.^4-8^ These findings suggest that the thalamus may be one of the hub regions that are part of being cognitively healthy during aging. Yet, it remains unclear whether the thalamus anatomy is independently associated with these cognitive domains or whether such an association is related to the correlation of the thalamus volume with other subcortical or frontal anatomy.

The thalamus is also involved in the circuits by exhibiting structural and functional connectivity with the hippocampus through the circuitry proposed by Papez *et al*.^3,5,6,9-13^ The hippocampus has dense white matter projections to the anterior thalamic nuclei through part of the Papez circuits, including the fornix.^14^ Studies have demonstrated the correlated volume in the thalamus and the hippocampus during aging.^3,15^ With repeat brain imaging, we may further expand the cross-sectional structural covariance findings by investigating whether thalamus volume loss precedes hippocampal atrophy and predicts subsequent hippocampal atrophy aside from the aging effect. Results from some studies suggest that thalamus atrophy precedes hippocampal atrophy in patients with mild cognitive impairments, but such evidence is lacking in general aging populations.^7,8^

The current study investigates the effect of thalamus volume on cognitive performance and the longitudinal structural relationship between the thalamus and the interconnected hippocampus in cognitively unimpaired community-dwelling older adults. We used T1-weighted (T1w) brain magnetic resonance imaging (MRI) data from the United Kingdom (UK) Biobank to segment the subcortical structures and estimate the volume. Aim-1 investigates the association between thalamus volume and cognitive performance. The primary cognitive domains of interest are executive function and psychomotor speed. We also include working memory as an additional domain of interest related to hippocampal function. With a relatively large cross-sectional sample, we test the hypothesis that thalamus volume will show an independent positive effect on executive function and psychomotor speed. A multivariable linear model was used to account for demographic and lifestyle variables (i.e. education, age, sex, alcohol, smoking, and sleeping) and regional/brain volume measures (i.e. frontal lobe gyrus GM volume, volume of nuclei within basal ganglia, volume of amygdala, and total brain volumes). Aim-2 explores the longitudinal relationship between the thalamus and hippocampal volume using a latent change score model (LCS) to test for a bi-directional association between baseline thalamus volume and hippocampal atrophy over an average of 2.3 years and vice versa. The hypothesis in Aim-2 tests whether the thalamus volume at baseline predicted the trajectory of hippocampal atrophy at follow-up. Additional sex-stratified analysis examined whether the LCS associations were influenced by age and sex, as men tend to show faster subcortical volume loss than women.^16^ As an exploratory analysis of Aim-2, we incorporated the fractional anisotropy (FA) of the fornix based on diffusion tensor imaging (DTI) into a structural connectivity model. The fornix is a major hippocampal projection towards the thalamus.^17^ We test whether thalamus and hippocampal volume loss was associated with the loss of the structural connectivity between them.

## Material and methods

### Ethics approval statements and patient consents

The UK Biobank project obtained ethical approval from the Northwest Multi-center Research Ethnic Committee, and each participant consented to participate. Participants who withdrew from the UK Biobank project until 2024-January was excluded from the current study.

### Participants and study design

**Figure 1:**
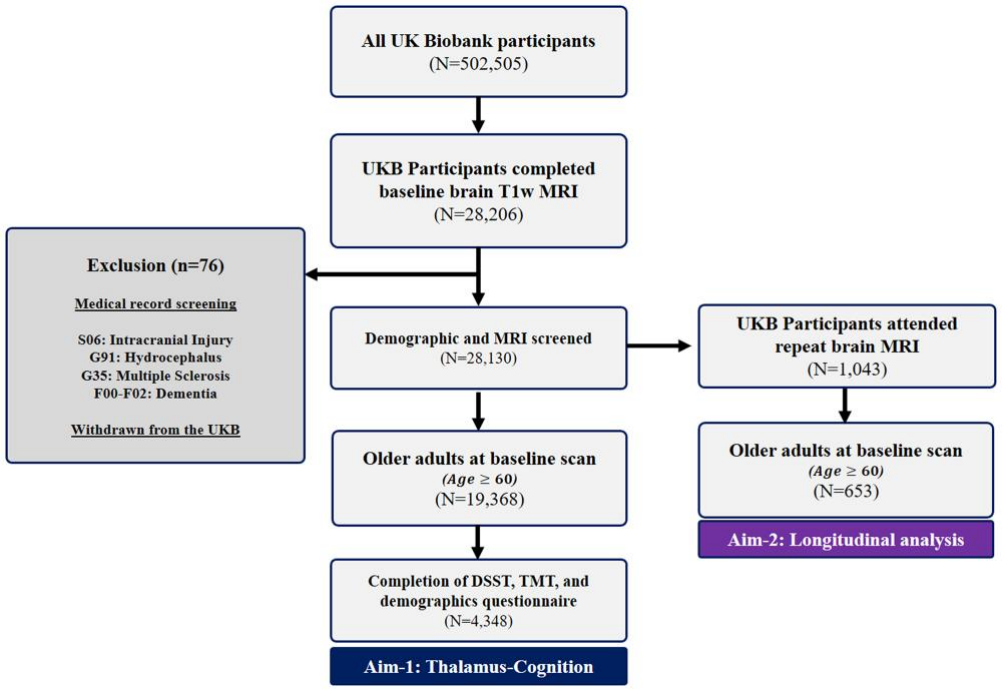
**Summary of the study design, inclusion, and exclusion criteria for the Aim-1 and Aim-2 analysis.** The medical record screening was based on the ICD-10 codes. DSST, Digit-symbol substitution test; TMT, Trail making test.

The study cohort comprised community-dwelling, cognitively unimpaired older adults from the UK Biobank. Cognitive impairment was defined based on medical records. Participants were excluded if they had a history of intracranial injury (ICD-10 code S06), hydrocephalus (ICD-10 code G91), multiple sclerosis (G35), or dementia (ICD-10 code F00-F02). We accessed the data release from an approved UK Biobank project (#55623). The UK Biobank project obtained ethical approval from the Northwest Multi-center Research Ethnic Committee, and each participant consented to participate. Participants who withdrew from the UK Biobank project until 2024-January were excluded from the current study. Figure 1 summarizes the inclusion and exclusion of the study cohort. The inclusion criteria were participants older than 60 at the baseline brain MRI scans and participants who had completed the DSST and TMT cognition tests. Participants were excluded if they were younger than 60 years old, did not complete brain MRI or cognition tests, or lacked demographic data. A total of 4348 older adults were identified for Aim-1, of whom 653 had follow-up MRI were included for Aim-2. Demographic data consisted of age, sex, education, smoking status, alcohol frequency, and hours of sleep.

### Cognitive measurements

We accessed the test results from the digital DSST and TMT cognition assessments. An example of a touch-screen-based DSST and TMT test is available at the UK Biobank data showcase: https://biobank.ndph.ox.ac.uk/showcase/refer.cgi?id=5022, https://biobank.ndph.ox.ac.uk/showcase/refer.cgi?id=104 and https://biobank.ndph.ox.ac.uk/showcase/refer.cgi?id=105. For the DSST, participants were presented with two tables. The first table showed the symbols and associated single-digit integers as a key, and the second only showed the symbols. The participants were asked to input the matched digit on the second table using a touchpad according to the key using a number pad on a touch screen within one minute. The number of correct symbol-digit matches made in one minute was used as the score for DSST. For the TMT part A, participants were presented with a set of digits from 1 to 25 and were instructed to click on them sequentially (e.g. 1–2–3– … −25). For TMT part B, participants were presented with a set of digits (1–13) and letters (A-L) and were instructed to click numbers and letters in an alternating way in ascending order (e.g. 1-A-2-B-…-13-L). Time to completion was used as the score for the TMT assessments. An exploratory factor analysis was then used to create a cognitive composite score from the standardized score of DSST number of correct matches, TMT-part A completion time, and TMT-part B completion time. The sign of the cognitive composite score was flipped so that a larger score corresponded to better performance, i.e. a higher DSST score and lower completion times for TMT-A and TMT-B. In addition, the numeric memory test (NMT) was treated separately as a measure of working memory performance. The NMT consists of a series of n-digit numbers for participants to remember. Starting at a 2-digit number (*n* = 2), the number appeared on a digital tablet screen and disappeared after 2 + 0.5*n* s. The number became one digit longer every time a participant remembered correctly, up to a maximum of 12 digits. The maximum digits the participants memorized correctly were recorded.

### MRI acquisition and processing

Participants were scanned on a Siemens Skyra 3T system with a 32-channel RF receive head coil. T1w images (208 × 256 × 256 matrix) were acquired during a 5-minute 3D MPRAGE scan sequence (TR = 2000 ms, TI = 880 ms, in-plane acceleration factor = 2, voxel size = 1 mm × 1 mm × 1 mm). We accessed defaced T1w MRI from the baseline and repeat imaging sessions. We relied on the FIRST toolbox from the FMRIB’s software library to segment the bilateral thalamus, hippocampus, amygdala, and basal ganglia, including putamen, pallidum, nucleus accumbens, and caudate nucleus regions. The total brain GM and white matter volume were estimated using the FSL-FAST toolbox. As an exploratory analysis of Aim-2, the mean FA of the fornix was estimated from DTI data (*b* = 1000 s/mm², 50 directions) using the DTIFIT toolbox. FA images were registered onto a standard-space white-matter skeleton through tract-based spatial statistics. Details of UK Biobank MRI sequence settings are available at: https://biobank.ndph.ox.ac.uk/showcase/refer.cgi?id = 1977.

### Statistical analyses

Demographic data were analyzed using the proportion or mean values plus standard deviation. For Aim-1, we tested for an association between the bilateral thalamus volume and the cognitive composite score using a multivariable linear model. Two classes of confounding variables were included in the linear model: (i) demographic and lifestyle variables, including age at scan, sex, age completed full-time education, smoking frequency, alcohol frequency, and hours of sleep; and (ii) brain volume covariates provided by the bilateral volume of the hippocampus, amygdala, putamen, pallidum, nucleus accumbens, caudate nucleus, frontal lobe gyrus GM volume, and brain total white matter volume. Frontal lobe gyrus GM volume was estimated as a sum of the regional GM volume of the superior frontal gyrus, middle frontal gyrus, inferior frontal gyrus pars opercularis, and inferior frontal gyrus pars triangularis. The association between the thalamus volume and the cognitive composite score and the raw test scores was assessed, and false-discovery rate correction was applied to *P*-values to control for multiple comparisons. The linear model is expressed as below. As a sensitivity analysis, we estimated TIV (eTIV), from total GM volume, total white matter volume, and total ventricular CSF volume as an additional covariate in the linear model. We also calculated the variance inflation factor (VIF) for each independent variable in Aim-1 to evaluate potential multicollinearity. A VIF value higher than 5 was set as the threshold for risk of collinearity. We also examined the association between thalamus and hippocampus volume with NMT scores using a separate linear model with the same confounding controls. The equation of the linear model is expressed below:


$$\begin{array}{rl} & \mathrm{Cognitive}\;\mathrm{composite}\;\mathrm{score}=\mathrm{Thalamus}\;\mathrm{volume}+\mathrm{Age}+\mathrm{Sex}\\ & +\mathrm{Age}\;\mathrm{completed}\;\mathrm{education}\;\;+\mathrm{Smoking}\;\mathrm{frequency}\\ & +\mathrm{Alcohol}\;\mathrm{frequency}+\mathrm{Hours}\;\mathrm{of}\;\mathrm{sleep}\\ & +\mathrm{Volume}\;\mathrm{of}\;\mathrm{hippocampus}+\mathrm{Volume}\;\mathrm{of}\;\mathrm{amygdala}\\ & +\mathrm{Volume}\;\mathrm{of}\;\mathrm{putamen}+\mathrm{Volume}\;\mathrm{of}\;\mathrm{pallidum}\\ & +\mathrm{Volume}\;\mathrm{of}\;\mathrm{nucleus}\;\mathrm{accumbens}+\mathrm{Volume}\;\mathrm{of}\;\mathrm{caudate}\\ & +\mathrm{Volume}\;\mathrm{of}\;\mathrm{frontal}\;\mathrm{lobe}\;\mathrm{grey}\;\mathrm{matter}\\ & +\mathrm{Total}\;\mathrm{brain}\;\mathrm{white}\;\mathrm{matter}\;\mathrm{volume} \end{array}$$

For Aim-2, we used a linear mixed-effect model with control of the time interval between scans to test for significant volume loss and hippocampus loss within the longitudinal samples. We used LCS models from the R package Lavaan version 0.6.17 (R version 4.3.3) to investigate the potential bidirectional longitudinal relationship between the thalamus and hippocampal volumes.^18^ We used a bivariate LCS to estimate a latent variable measuring the atrophy volume over time. A collection of linear regression and covariance measurements were included in the LCS model. The LCS model permits the simultaneous and bidirectional estimation of the association between the trajectory of the hippocampus/thalamus volume over time.^18^ Using a latent variable also helps control random error from a direct subtraction.^18,19^ Two latent change score variables were estimated from baseline and follow-up volume estimates to map the thalamus volume loss (ΔThal) and hippocampal volume loss (ΔHipp) over time. The sign of the change score was flipped to keep the atrophy volume as a positive value. The bivariate LCS model was used to estimate the association ($\gamma_{T\to dH}$) between the baseline thalamus volume $V(\mathrm{Thal}{)}_{T1}$ and ΔHipp, and the association ($\gamma_{H\to dT}$) between the baseline hippocampal volume $V(\mathrm{Hipp}{)}_{T1}$ and ΔThal. The LCS model also measures the association ($\beta_{H}$) between $V(\mathrm{Hipp}{)}_{T1}$ and ΔHipp, and the association ($\beta_{T}$) between $V(\mathrm{Thal}{)}_{T1}$ and ΔThal. The bivariate LCS model also estimated the covariance between the baseline hippocampus and thalamus volume $\left(\phi_{HT}\right)$, and the covariance between the volume loss of the hippocampus and thalamus over time $\left(\rho_{HT}\right)$. The regression equations of the bivariate LCS model are expressed below.


$$\begin{array}{rl} & \mathrm{Bivarate}\;\mathrm{LCS}\\ & \{\begin{array}{c} \;\Delta Hipp=\beta_{H}\times V{\left(Hipp\right)}_{T1}+\gamma_{T\to dH}\times V{\left(Thal\right)}_{T1}+\epsilon \\ \;\Delta Thal=\;\beta_{T}\times V{\left(Thal\right)}_{T1}+\gamma_{H\to dT}\times V{\left(Hipp\right)}_{T1}+\epsilon \end{array} \end{array}$$

We tested sex effects using a multigroup bivariate LCS model.^18^ We also ran a sensitivity analysis using linear models to validate the directionality of thalamus–hippocampus associations observed in the bivariate LCS model with extra confounding variables, adjusting age, sex, total brain volume, smoking, alcohol frequency, and years between the baseline and repeat imaging visit.

To examine the age effect on the thalamus-hippocampal bidirectional association, we adopted a multivariate LCS model by adding a third change score to reflect age at baseline and the time between baseline and follow-up MRI scans. Hence, the between-subject differences in time between scans were represented as an age change score (ΔAge). The regression equations of the multivariate LCS model are expressed below.


$$\begin{array}{rl} & \text{Multivariate LCS}\\ & \{\begin{array}{c} \;\Delta \mathrm{Hipp}=\beta_{H|\mathrm{Age}}\times V{\left(\mathrm{Hipp}\right)}_{T1}+\gamma_{T\to dH|\mathrm{Age}}\times V{\left(\mathrm{Thal}\right)}_{T1}\\ \;\;+\;\gamma_{\mathrm{Age}\to dH}\;\times \;\mathrm{Age}+\epsilon \\ \;\Delta \mathrm{Thal}=\beta_{T|\mathrm{Age}}\times V{\left(\mathrm{Thal}\right)}_{T1}+\gamma_{T\to dT|\mathrm{Age}}\times V{\left(\mathrm{Hipp}\right)}_{T1}\\ \;\;+\;\gamma_{\mathrm{Age}\to dT}\;\times \;\mathrm{Age}+\epsilon \end{array} \end{array}$$

For the structural connectivity analysis, we tested whether baseline fornix FA at the baseline scan was associated with hippocampus and thalamus volume at the follow-up scan. Age, sex, total brain volume, smoking status, frequency of alcohol consumption, hippocampus and thalamus volume at baseline, and the years between the baseline and repeat imaging visit were included as covariates.

We performed a series of sensitivity analyses to investigate potential selection bias in the Aim-2 sample due to an observable drop in sample size compared with the Aim-1 sample. Analyses included: (i). Estimating propensity scores to create a subset of well-matched Aim-2 and Aim-1 samples; (ii). Full Information Maximum Likelihood (FIML) approach; and (iii). Multiple Imputation (MI) approach.^20^ An attrition table was created to compare the baseline characteristics between Aim-1 and Aim-2 samples. Propensity scores were estimated using the R library MatchIt (ver 4.1.7). Smoking status, alcohol frequency, diabetes diagnosis, and body mass index were used to estimate propensity scores for each participant. A 1:1 nearest-neighbor matching without replacement was conducted to match samples in Aim-2 to Aim-1, applying a strict caliper of 0.001 on the propensity score. This procedure resulted in a subset of longitudinal participants well-matched to a larger cross-sectional sample. A sensitivity analysis was performed by running the bivariate LCS model using the matched longitudinal sample. FIML and MI methods were employed with the bivariate LCS models to address missing data under the assumption of missing-at-random. For the FIML analysis, we applied the bivariate LCS models to the full Aim-1 sample, comprising a subset of participants with both baseline and follow-up MRI data, and the remaining participants with missing follow-up data. The FIML analysis was performed using the missing = ‘fiml’ option in the Lavaan R package. For MI analysis, we used the R libraries Mice (version 3.18.0, Predictive mean matching) and Jomo (version 2.7.6, linear mixed-effects model imputation). Both methods impute missing follow-up thalamus and hippocampus volumes for the Aim-1 sample. The imputation models included baseline brain volumes, age, and biological sex as predictors in the linear mixed effect model. A total of 40 imputed datasets were generated from each method and subsequently analyzed using the bivariate LCS model framework. We also investigated the impact of two automated segmentation methods on the hippocampus and thalamus volume estimates. A total of 125 paired (baseline and follow-up) cases were selected at random from the Aim-2 sample. FSL (‘fsl_anat’) and the longitudinal version of FreeSurfer (‘recon_all -long’, version 6.0) were run. Bland-Altman plots were generated to visualize possible systematic differences in the volume estimates. We examined the number of participants who showed volumetric estimate differences >1.96 standard deviations (SD) limits of agreement (LOA).

## Results

**Figure 2:**
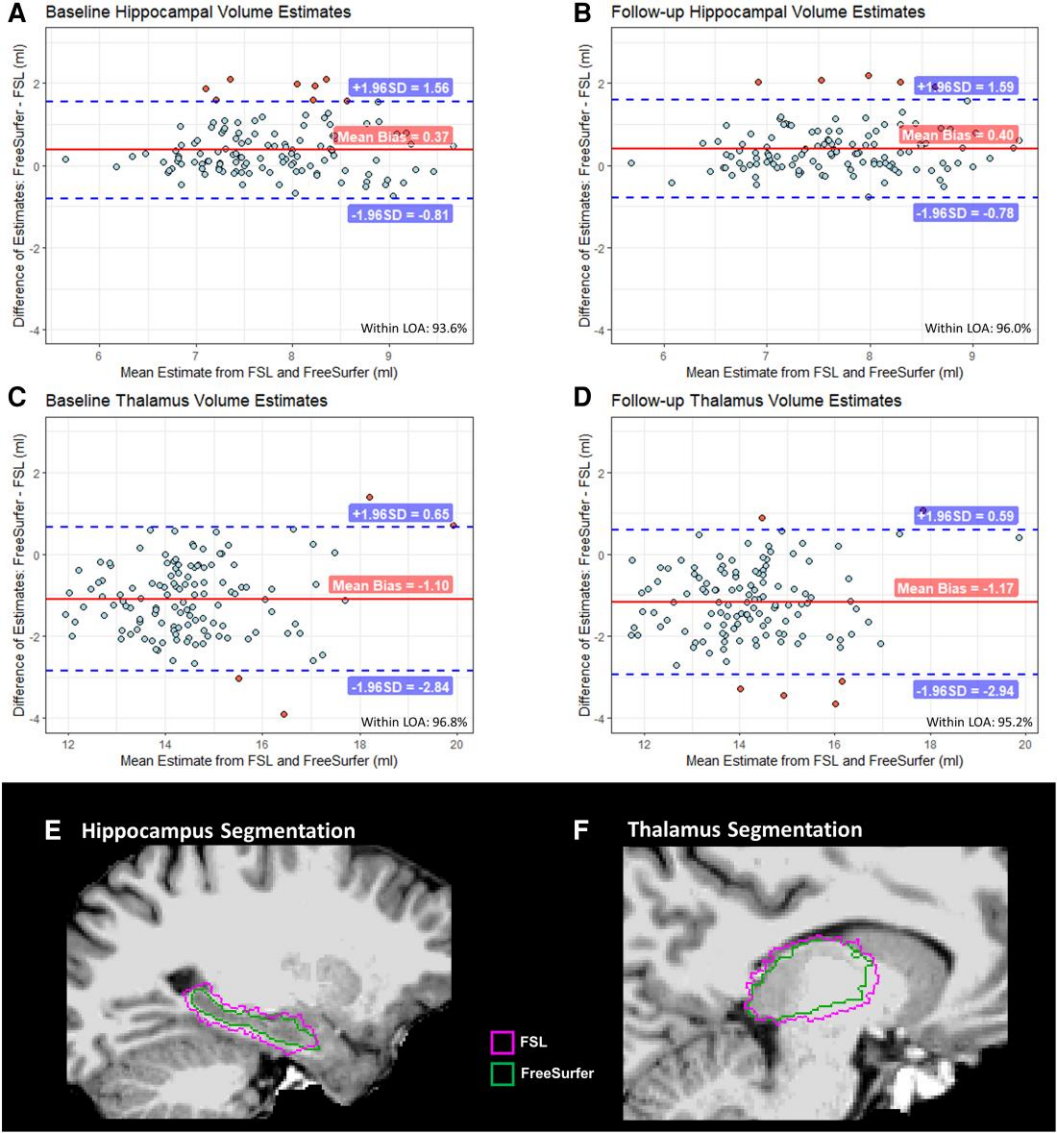
**Comparison of hippocampal and thalamic segmentation between automatic segmentation pipelines.** (**A–D**) shows Bland-Altman plots comparing hippocampal and thalamic volume estimates (mL) using FSL and FreeSurfer segmentation pipelines at baseline and follow-up scans from 125 randomly selected participants. Each data point represents the difference between FreeSurfer and FSL estimates, plotted against the mean volume estimates. The solid lines indicate the average difference (Mean bias, mL) of the volume estimates between FreeSurfer and FSL pipeline. The dashed lines represent the 95% LOA (Mean bias ± 1.96 SD). The percentage of data points falling within the LOA is reported. (**E**, **F**) shows representative examples of hippocampal (**E**) and thalamic (**F**) segmentations from one randomly selected participant. FSL, FMRIB Software Library; LOA, Limits of Agreement; SD, Standard Deviation.

Demographic details and cognitive test scores were summarized in Table 1. The cognitive composite score (Min = −5.21, Median = 0.14, Max = 1.89) was positively correlated with the numbers of DSST correct matches (*r* = 0.66) and negatively correlated with time completing TMT-A (*r* = −0.78) and TMT-B (*r* = −0.93), explaining 49.9% of the total variance. A higher positive score hence equated to better cognitive performance. The consistency analysis revealed that 93.6% of the baseline and 96.0% of the follow-up hippocampal volume estimates were within ±1.96 SD LOA. For the thalamus volume estimates, agreement was observed for 96.8% at baseline and 95.2% at follow-up measurement. (Figure 2).

**Table 1:** Summary of the participant demographics and cognitive test scores.

Demographics and lifestyle variables	(Mean *μ* ± standard deviation *σ*, or proportion %)
Age at scan date (years)	67.9 ± 4.8
Sex	Men: 1991 (45.4%)
	Women: 2357 (54.6%)
Age completed full time educations (years)	17.2 ± 2.3
Ethnic background	White: 4300 (98.9%)
	Black: 9 (0.2%)
	Asian: 13 (0.3%)
	Others: 26 (0.6%)
Smoking status	Never: 2335 (53.7%)
	Past: 1783 (41.0%)
	Current: 230 (5.3%)
Alcohol frequency score	2.73 ± 1.42
1 = Daily	
2 = 3–4 times a week	
3 = 1–2 times a week	
4 = 1–3 times a month	
5 = Special occasions only	
6 = Never	
Self-reported sleep duration (hours)	7.2 ± 1.0

Cognitive test scores	(Mean *μ* ± standard deviation *σ*)
TMT Part A time (s)	41.2 ± 14.8
TMT Part B time (s)	71.3 ± 25.6
DSST numbers of correct matches	19.0 ± 4.6

Continuous variables are reported as mean ± standard deviation. DSST, Digit symbol substitution test; TMT, Trail making test.

A summary of brain MRI estimates were included in Table 2. For Aim-1, the thalamus volume was positively associated with the cognitive composite score ($\beta_{\mathrm{Thal}}=0.069\pm 0.017,\;P<0.001,\;R^{2}=0.09$) after controlling for demographic, lifestyle, and brain volume covariates (Table 3, Supplementary Fig. 2). In terms of the covariates, we observed that the frontal lobe gyrus GM volume ($\beta_{\mathrm{FL}}=0.008\pm 0.003$, *P* = 0.007) and age completed education ($\beta_{\mathrm{edu}}=0.030\pm 0.006$, *P* < 0.001) also showed positive associations with the cognitive composite score. On the other hand, age ($\beta_{\mathrm{Age}}=-0.039\pm 0.003$, *P* < 0.001), and amygdala volume ($\beta_{\mathrm{Amyg}}=-0.069\pm 0.033$, *P* = 0.037) showed negative associations with the cognitive composite score. The cognitive composite score was not significantly associated with smoking status, alcohol frequency, and hours of sleep, nor the volume of the hippocampus, putamen, pallidum, nucleus accumbens, and caudate nucleus (*P* > 0.05). From NMT, we found that the maximum number of digits people memorized was positively correlated with thalamus volume (β=0.10±0.05,P=0.03) and frontal lobe GM volume (*β* = 0.03 ± 0.01, *P* = 0.001). Hippocampal volume was not significantly associated with the NMT score (*β* = 0.04 ± 0.05, *P* = 0.43). The overall model fit (*R*² = 0.027) was weak. All independent variables in the linear models showed VIF < 5.0. Including eTIV in the linear model did not alter the significant association between thalamus volume and the cognition composite score (*β* = 0.06 ± 0.02, *P* = 0.001), while eTIV showed a higher VIF (5.54) compared with the total WM volume (3.47), suggesting a moderate risk of collinearity. (Supplementary Table 2) Results from additional linear models that examined the association between the thalamus volume and each independent test score and NMT scores are available in Supplementary Table 1.

**Table 2:** Summary of the participant brain regional volumetric estimates.

Cross-sectional sample for thalamus-cognition analysis (Aim-1, *n* = 4 348)	
Brain MRI estimates	(Mean *μ* ± standard deviation *σ*)
Total Brain GM + WM volume (L)	1.14 ± 0.11
Total Brain WM volume (L)	0.54 ± 0.06
Estimated Total Intracranial volume (L)	1.17 ± 0.11
Thalamus volume (mL)	14.95 ± 1.34
Hippocampus volume (mL)	7.52 ± 0.85
Amygdala volume (mL)	2.46 ± 0.43
Caudate volume (mL)	6.85 ± 0.81
Putamen volume (mL)	9.36 ± 1.07
Pallidum volume (mL)	3.49 ± 0.46
Nucleus Accumbens volume (mL)	0.83 ± 0.20
Frontal lobe GM volume (mL)^a^	48.9 ± 5.83

Longitudinal samples for thalamus-hippocampal LCS model (Aim-2, *n* = 653, Time between scans: 817.6 ± 45.4 days)		
Brain MRI estimates	Baseline scan (*μ* ± *σ*)	Follow-up scan (*μ* ± *σ*, *T*-value^b^)
Thalamus volume (mL)	15.09 ± 1.40	14.88 ± 1.39 (*T* = 12.3)*
Hippocampus volume (mL)	7.62 ± 0.89	7.47 ± 0.88 (*T* = 19.3)*

Continuous variables are reported as mean ± standard deviation. For Aim-2 volumetric estimates, linear mixed effect models were used tested for a volume difference between the baseline and follow-up after accounting for between-subject differences in the time between scans (* denotes *P* < 0.05). DSST, Digit symbol substitution test; GM, Grey matter; TMT, Trail making test; WM, White matter.

^a^Frontal lobe GM volume is a sum of the GM volume of the superior frontal gyrus, middle frontal gyrus, inferior frontal gyrus pars opercularis, and inferior frontal gyrus pars triangularis.

^b^ *T*-value from the session effect in the linear mix effect model: Volume = Session + Time between scans + (1|Patient ID).

^*^Represents significantly lower regional brain volume in follow-up scan (*P* < 0.05).

**Table 3:** Summary of the multivariable linear model showing the significant association between thalamus volume and cognitive composite score.

Outcome variable		
Cognitive composite score (Min = −5.21, Median = 0.14, Mean = 0, Max = 1.89)		
Predictors and confounders	*β* ± SE	*P*-value
Thalamus volume	0.069 ± 0.017	<0.001 *
Frontal lobe gyrus GM volume	0.008 ± 0.003	0.007 *
Age	−0.039 ± 0.003	<0.001 *
Sex: Men	−0.053 ± 0.033	0.112
Age completed full time education	0.030 ± 0.006	<0.001 *
Smoking: past/current smoker	0.020 ± 0.022	0.365
Alcohol frequency	−0.034 ± 0.009	0.714
Self-reported sleep duration	−0.007 ± 0.013	0.565
Total brain white matter volume	−0.004 ± 0.001	0.253
Hippocampus volume	0.024 ± 0.020	0.228
Caudate volume	−0.015 ± 0.020	0.453
Putamen volume	0.035 ± 0.018	0.052
Pallidum volume	0.004 ± 0.036	0.901
Amygdala volume	−0.069 ± 0.033	0.037 *
Nucleus Accumbens volume	−0.001 ± 0.080	0.939

Beta weights and standard errors were estimated, and variables with statistically significant (*P* < 0.05) association with the cognitive composite score were marked with * sign beside the *P*-values. The intercept was 0.817 ± 0.326 (*P* = 0.01). The model adjusted *R*^2^ was 0.09. GM, Grey matter; SE, Standard errors; WM, White matter.

^*^Represents significantly lower regional brain volume in follow-up scan (*P* < 0.05).

**Figure 3:**
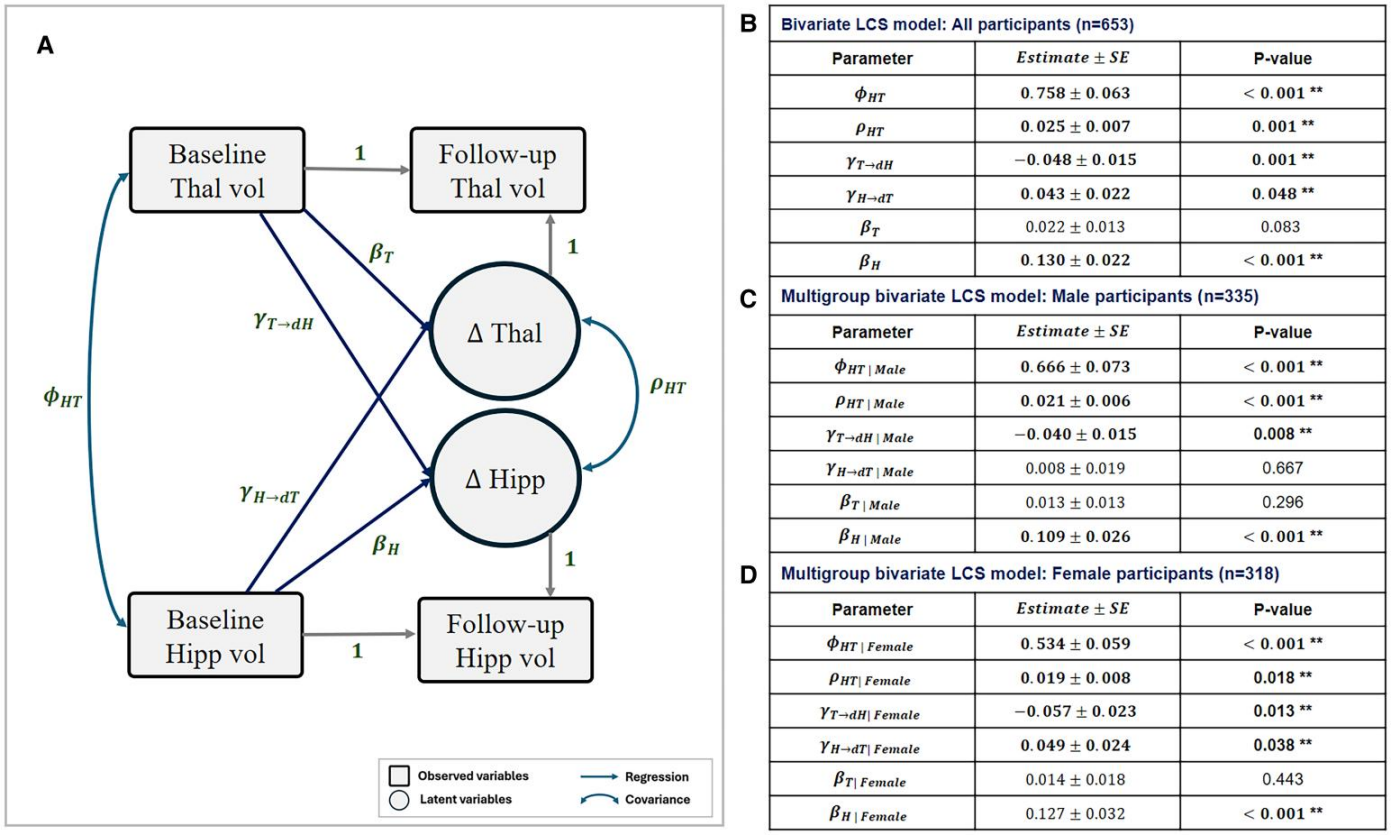
**Summary of the bivariate LCS model fitting.** (**A**) Shows the path diagram with the parameter estimates. ΔThal and ΔHipp are two latent variables whose positive values indicate volume loss over time. (**B**) Shows the estimation of the covariance and regression coefficients for the bivariate LCS model. (**C, D**) shows the coefficient and covariance estimates from sex-stratified multigroup bivariate LCS models. A regression path labelled ‘1’ indicates the regression coefficient was fixed to 1. Significant associations and covariances are marked with (**) sign. Hipp, Hippocampus; LCS, Latent Change Score Model; Thal, Thalamus; Vol, Volume.

For Aim-2, the bilateral thalamus and hippocampus volumes were lower at follow-up compared with baseline (Table 2). Figure 3 summarizes the results from the bivariate LCS models. The latent change scores were denoted as ΔHipp (Mean = 0.155, SD = 0.136) and ΔThal (Mean = 0.213, SD = 0.132), represented an estimation of atrophy volume, where positive value indicated greater volume loss over time. The baseline thalamus volume and the baseline hippocampus volume showed positive covariance ($\phi_{HT}=0.76\pm 0.06$, *P* < 0.001). The ΔHipp and ΔThal change scores also showed significant positive covariance ($\rho_{HT}=0.03\pm 0.01$, *P* = 0.001). We observed a significant, bidirectional longitudinal relationship between thalamic and hippocampal volumes. Thalamus volume at baseline showed a negative association with ΔHipp ($\gamma_{T\to dH}=-0.048\pm 0.015$, *P* = 0.001), while hippocampal volume at baseline showed a positive association with ΔThal ($\gamma_{H\to dT}=0.043\pm 0.022$, *P* = 0.048). In the exploratory structural connectivity analysis, we found that fornix mean FA was both positively correlated with hippocampal volume (β=0.62±0.17,P<0.001) and thalamus volume (β=0.48±0.16,P=0.002) at follow-up scans. (Supplementary Table 4). Sensitivity analysis with additional confounding variables showed that baseline thalamus volume robustly predicted hippocampal atrophy (Supplementary Table 5).

In the sex-stratified multigroup bivariate LCS models (Fig. 3), the significant positive covariance terms ($\phi_{HT}$ and $\rho_{HT}$) remained significant in both men and women. There was no change in the $\beta_{T}$ and $\beta_{H}$ as well. Women showed significant bidirectional relationships that aligned with the non-stratified BLCS model ($\gamma_{T\to dH|\mathrm{Female}}=-0.057\pm 0.023$, *P* = 0.01; $\gamma_{H\to dT|\mathrm{Female}}=0.049\pm 0.024$, *P* = 0.038). Men showed a significant unidirectional relationship between the thalamus and hippocampus, namely baseline thalamus volume significantly predicted ΔHipp ($\gamma_{T\to dH|\mathrm{Male}}=-0.040\pm 0.015$, *P* = 0.008), yet baseline hippocampus volume was not significantly associated with ΔThal ($\gamma_{H\to dT|\mathrm{Male}}=0.008\pm 0.019$, *P* = 0.667). Parameter fits of the multivariate LCS model were available in Supplementary Fig. 1. Age at baseline scan showed negative covariance with baseline hippocampal volume ($\phi_{H,\mathrm{Age}}=-1.10\pm 0.17$, *P* < 0.001) and baseline thalamus volume ($\phi_{T,\mathrm{Age}}=-1.63\pm 0.27$, *P* < 0.001), yet the time interval between the MRI scans (mean = 2.26, IQR = 0.33) did not show significant covariance with ΔHipp and ΔThal. Age at baseline was significantly associated with hippocampal volume loss ($\gamma_{\mathrm{Age}\to dH}=0.013\pm 0.003$, *P* < 0.001), while age at baseline was not associated with thalamus volume loss. After adding age as a confounding variable, the bidirectional relationship between the longitudinal change of hippocampal volume and thalamus volume remained significant ($\gamma_{T\to dH|\mathrm{Age}}=-0.042\pm 0.011$, *P* < 0.001; $\gamma_{H\to dT|\mathrm{Age}}=0.048\pm 0.017$, *P* = 0.004).

We conducted sensitivity analyses to evaluate the potential selection bias introduced by the drop in sample size from Aim-1 to Aim-2. An attrition summary was included in Supplementary Table 3. By estimating the propensity scores, a total of *N* = 539 Aim-2 participants (82.3%) were matched to the Aim-1 sample. The estimated effect of baseline thalamus volume on hippocampal change remained consistent with the full Aim-2 sample ($\gamma_{T\to dH}=-0.041\pm 0.012,P=0.001$). MI analysis using two MI models both showed that baseline thalamus volume significantly explains the dHipp change score. Predictive mean matching method: $\gamma_{T\to dH}\;=-0.052\pm 0.005,P<0.001$; Linear mixed effect modeling method: ($\gamma_{T\to dH}=-0.034\pm 0.014,P=0.03$). FIML analysis did not yield a significant association between thalamus volume and dHipp ($\gamma_{T\to dH}=$  −0.04±0.02,P=0.09).

## Discussion

This study investigated thalamus anatomy in relation to cognitive performance and demonstrated the bidirectional longitudinal volumetric relationship between the structurally connected thalamus and hippocampus in community-dwelling older adults from the UK Biobank. Thalamus volume showed a positive association with psychomotor speed and executive functioning after controlling for demographics, regional, and global neuroimaging estimates. Using LCS models, we observed a pattern whereby larger thalamus volume at baseline predicted slower hippocampal volume loss over an average of 2.3 years in both men and women. Sex-stratified analysis also revealed differences between men and women participants in such bidirectional patterns. The results were further supported by explicitly accounting for age effect in a multivariate LCS model. These findings add new insight into the contribution of the thalamus to explain aging and cognition.

Thalamus volume correlated positively with the cognitive composite score estimated from the DSST, TMT-A, and TMT-B. Good performance on these tests requires intact psychomotor speed, visual perception, attention, and executive function.^21,22^ Past studies showed good validity of such touchscreen-based TMT and DSST.^23^ The thalamus is involved in multiple neuronal circuits, including the mammillothalamic tract, the superior spinothalamic tract, and the frontostriatal-thalamic tract, forming connections with multiple primary sensory regions.^10,24^ Since the thalamus is predominantly GM, the volume estimate thus likely reflects the gross density of the neuron cell body and contributes to cognitive performance during the DSST and TMT tests. Past literature on thalamus anatomy focused on patients with multiple sclerosis and neurodegenerative diseases, and few studies have investigated the thalamus-cognition association in healthy aging individuals. With the large current sample, we demonstrated a robust association between the thalamus and cognition. From the multiple linear models, we also observed that aside from the age, education, and sex effect on cognitive performance, that aligns with the past literature.^25^ Hippocampal volume was not significantly associated with NMT scores. There are several possible interpretations. Performances from NMT are best interpreted as a measure of working memory rather than long-term episodic memory, in which the hippocampus is less involved when compared with the prefrontal cortex.^25^ One past study also reported that the UK Biobank digital version of NMT showed relatively low validity compared with the in-person WAIS-IV Digit Span tests.^23^

From the longitudinal LCS models, we observed positive covariance between baseline thalamus volume and baseline hippocampal volume. There was also positive covariance between the thalamus and hippocampal volume changes over time. These findings align with the literature on the structural covariance networks within the hippocampus and thalamus, demonstrating the aging effect on general brain atrophy.^26^ In the whole longitudinal sample, we observed a bidirectional relationship between the longitudinal change of the thalamus and hippocampal volume. Larger thalamus volume at baseline predicts slower hippocampal volume loss over time. The structural connectivity analysis revealed that the structural disconnection between the thalamus and hippocampus may contribute to the volume loss in both structures over time. Future studies will investigate the functional connectivity between the thalamus and the hippocampus. The longitudinal findings remained significant after accounting for a possible selection and/or retention bias in the longitudinal sample. The propensity score analysis showed that restricting the analysis to a subsample of Aim-2 participants matched to the larger Aim-1 cohort did not alter the bivariate LCS model estimates. The MI analysis supported the association between baseline thalamus volume and hippocampal atrophy. While the FIML analysis did not reach statistical significance, the estimated direction and magnitude were comparable. These converging findings suggest that this relationship is likely generalizable to the broader population. We also conducted consistency analyses of volumetric estimates across FSL and FreeSurfer segmentation pipelines. The vast majority of hippocampal and thalamic volume estimates fell within one standard deviation, indicating that the choice of the segmentation algorithm was consistent for the two main brain regions considered in this study.

Using the multivariate LCS models to control for age at baseline and time between scans, we demonstrated that the bidirectional association between the hippocampus and thalamus remained significant. Several explanations help to support these findings. Atrophy in the thalamus can be directly associated with impaired reciprocal communication with the hippocampus and affect the hippocampal plasticity due to reduced active task engagements. In preclinical models, lesions in the thalamus were associated with reduced acetylcholine levels in the hippocampus and impaired learning ability.^27^ In clinical samples, infarction in the medial and anterior portion of the thalamus was associated with memory deficit and hippocampal atrophy.^28,29^ The thalamus may show higher vulnerability than the hippocampus in response to chronic diseases during aging, such as hypertension, which could significantly impact thalamus anatomy more than the hippocampus.^30^ Sensitivity analyses with extra confounder controls support a directional association in which baseline thalamus volume explains hippocampal atrophy.

The hippocampal volume showed a positive association with thalamus atrophy. A sex-stratified bivariate LCS model further elucidates that such bi-directional association was only present in women participants. For men, we observed a unidirectional association that thalamus volume at baseline predicts hippocampal volume loss, but not the other way around. The counterintuitive observation suggests that thalamus atrophy had been high when people still had intact hippocampal anatomy. Clinical evidence supports such interpretation, as thalamus atrophy has been reported in patients with mild amnestic cognitive impairments prior to hippocampal atrophy.^7^ The volume loss of the thalamus had been high prior to the hippocampus showing significant atrophy and had slowed down when hippocampal atrophy accelerated. We observed such a bidirectional association only in women, not in men. Possible explanations include sex differences in the brain circuits and the role of sex hormones. First, one diffusion MRI study reports that men showed higher FA in the thalamus than women, indicating sex differences in the structural integrity and organization within the thalamus.^31^ Reduced structural integrity of the thalamus can lead to thalamus volume loss at an earlier age in women compared with men. Second, estrogen is neuroprotective, and there is a greater variation in these levels among women than men.^32,33^ Unfortunately, estrogen data were unavailable in the current study to support further investigation.

This study has several limitations. First, we used an automatic hippocampus/thalamus segmentation algorithm consistent with other UK Biobank projects and conducive to large neuroimaging samples. Other approaches were available, but such a comparison of methods is beyond the scope of the current project.^34^ Second, we did not attempt to subdivide the subcortical anatomy, e.g. the thalamic nuclei or hippocampal subfields. Further research using higher main magnetic field MRI would be relevant to take advantage of the higher signal-to-noise ratio relative to 3 Tesla, thereby pursuing ultra-high spatial resolution imaging of subcortical anatomy for shape analysis. Third, the longitudinal analysis produced novel findings; however, we noted that the sample size dropped from the cross-sectional to the longitudinal aim, which may have introduced retention bias in Aim-2. It is important to note some of the demographic details for the current study. Namely, the cohort was predominantly white, mostly midlife or older, and cognitively unimpaired adults. There is a potential that the current findings may not be generalized to other populations or ethnic groups; however, we note that evidence of such volume association between the hippocampus and thalamus was observed in several other subsamples, including Asian and American.^8,35,36^ Future research is warranted to investigate such anatomical relationships in different populations to enhance generalizability. In Aim-1, our cognitive composite score were based on a 5-min touchscreen digital test (TMT and DSST), while long-term memory assessment of hippocampal function was not available in the current study. It is also important to note that anatomical variability may not explain other factors that could contribute to thalamic–hippocampal connectivity. For instance, GABA, acetylcholine, and serotonin neurotransmitters are associated with the excitability of both thalamic and hippocampal neurons, which could modulate neuronal activity and/or synaptic plasticity.^37,38^ Furthermore, regional volume estimates may also fail to account for pathological features, such as cerebral small vessel diseases that impact subcortical brain regions.^39^ Future work is warranted to address relationships between anatomy, function, and neurochemistry, which is possible with MRI acquisitions that yield spectroscopic and perfusion information.

The thalamus plays an important role in cognition during healthy aging. Thalamus volume may predict atrophy in the interconnected brain regions. Using repeat T1w MRI and a large sample of community-dwelling older adults from UK Biobank, the current study revealed a robust association between the thalamus volume and the cognitive performance in psychomotor speed and executive functioning in older adults. The study used LCS models to reveal a bidirectional longitudinal relationship between the volume loss of the thalamus and hippocampus in the aging population. Larger thalamus volume at baseline predicted slower hippocampal volume loss in both men and women, while hippocampal volume at baseline predicted faster thalamic volume loss. Thalamus volume loss had been high among individuals with large hippocampal volume, particularly in women participants. These findings add to the current literature on the thalamus-age-cognition relationship, revealing that thalamus volume predicts hippocampal volume loss and underscores the thalamus as an important marker of cognitive health during aging.

## Supplementary Material

fcaf334_Supplementary_Data

## Data Availability

This research has been conducted using the UK Biobank resource under application number 55623. UK Biobank provides the CSV and MRI data download. T1w images are processed using the open-sourced software FSL-FIRST. (https://fsl.fmrib.ox.ac.uk/fsl/fslwiki/FIRST) The full MRI scan protocols are available via UK Biobank (https://biobank.ndph.ox.ac.uk/showcase/refer.cgi?id=2367). All URLs and modeling equations of the key processing pipelines were attached to the manuscript and the Supplementary material.
