# Supplementary material for: Thalamus anatomy predicts cognitive performance and hippocampal atrophy : in aging adults: a UK Biobank study
Source: Brain Commun. 2025 Sep 10;7(5):fcaf334. doi: 10.1093/braincomms/fcaf334 (PMC12455194; doi:10.1093/braincomms/fcaf334)
Supplement: fcaf334_Supplementary_Data [file fcaf334_supplementary_data.docx]

**Supplementary Materials**


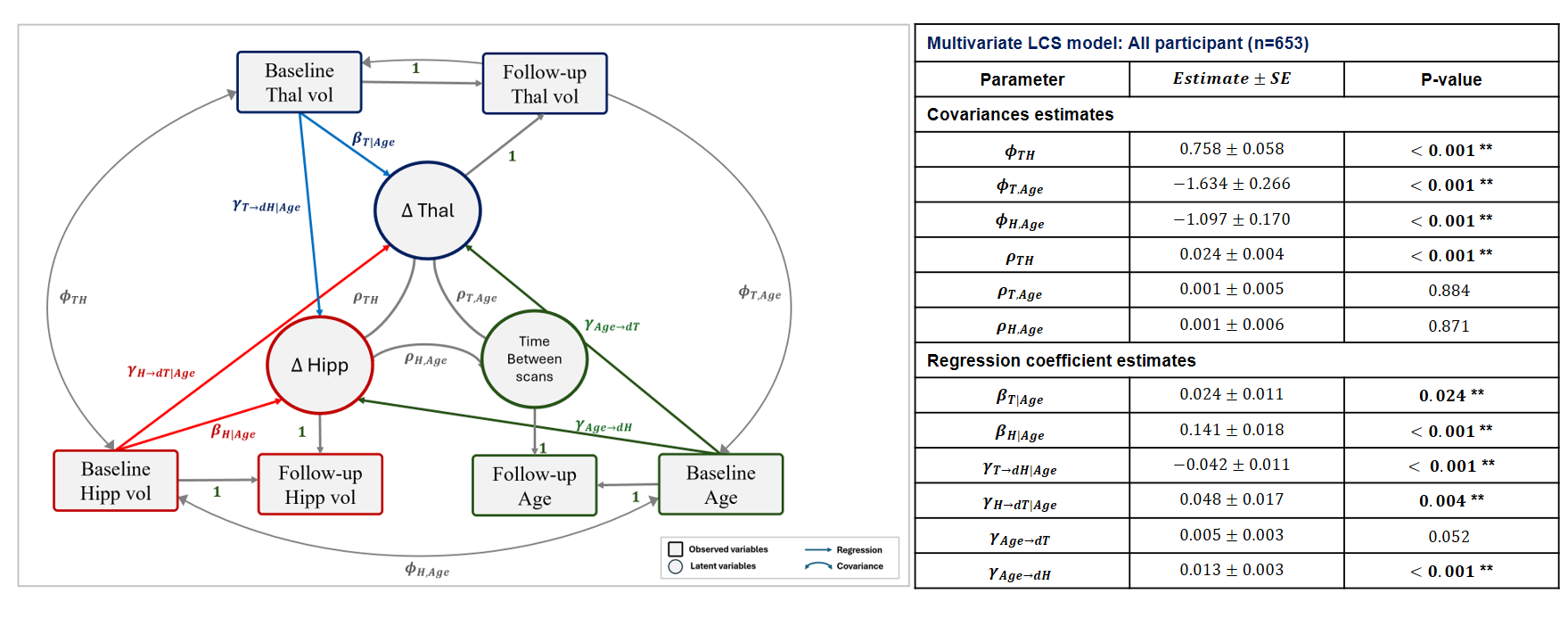


**Supplementary Figure 1**. Summary of the multivariate LCS model fitting. The left panel showed a path diagram with parameter estimates. A latent variable was marked as a circle, and an observed variable was marked as a rectangle. The right panel showed the estimation of the covariance and regression coefficients. A path labeled “1” indicates a regression with the coefficient fixed to 1. Significant P-values were marked with an (**) sign. Thal: Thalamus, Hipp: Hippocampus.


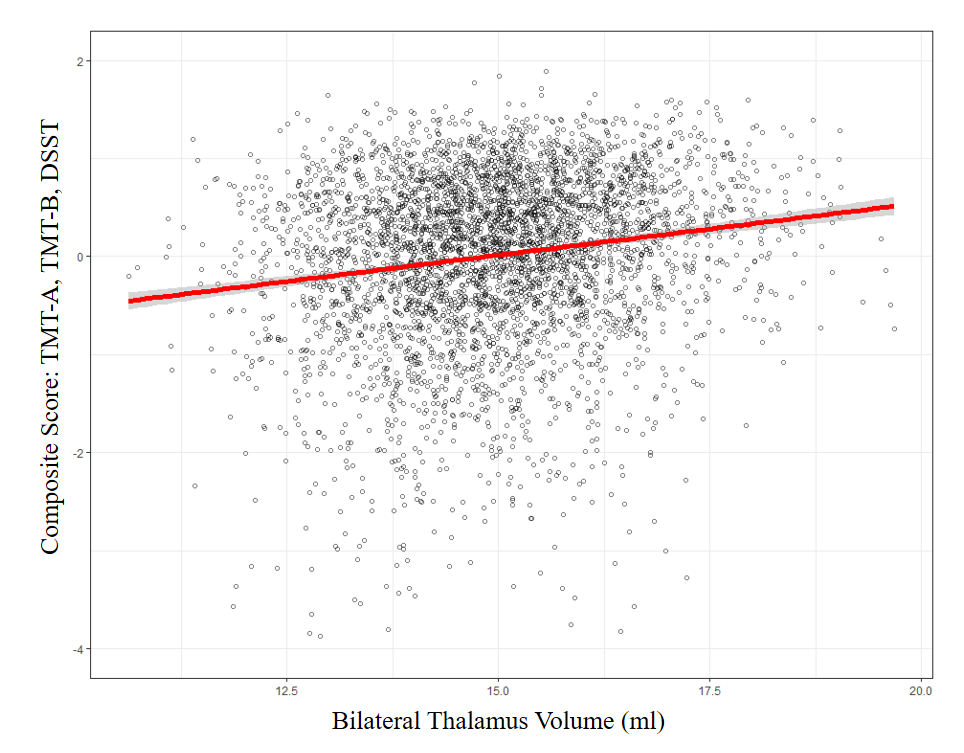


**Supplementary Figure 2.** Scatterplot showing a significant positive linear association between bilateral thalamus volume (ml) and the cognitive composite score estimated from the time completing the TMT-A, the TMT-B, and number of correct matches in the DSST. The association was evaluated using simple linear regression (β=0.11±0.01, T = 11.2, P < 0.001) and Pearson’s correlation (Pearson’s r = 0.17, T = 11.2, P < 0.001). Each data point represents the bilateral thalamic volume and the corresponding composite cognitive score from one participant. TMT: Trail Making Test, DSST: Digit-Symbol Substitution Test.

|  | **Cognitive measurements**  **(**$\boldsymbol{\beta\pm SE}$**)** | | | | |
| --- | --- | --- | --- | --- | --- |
|  | **Aim-1 Cognitive Composite Score** | | | **Extra Test I** | **Extra Test II** |
| **Predictors and confounders** | **DSST Correct Match** | **TMT-A Time (s)** | **TMT-B Time (s)** | **TMT B-A Time (s)** | **Max Digit Remember NMT** |
| Thalamus volume | $\boldsymbol{0.37\pm0.09}$ ****** | $-0.55\pm0.30$ | $\boldsymbol{-1.90\pm0.50}$ ****** | $\boldsymbol{-1.35\pm0.42}$ ****** | $\boldsymbol{0.10\pm0.05}$ ****** |
| Age | $\boldsymbol{-0.23\pm0.02}$ ****** | $\boldsymbol{0.39\pm0.05}$ ****** | $\boldsymbol{0.98\pm0.08}$ ****** | $\boldsymbol{-0.87\pm0.13}$ ****** | $\boldsymbol{-0.04\pm0.01}$ ****** |
| Sex: Men | $\boldsymbol{-0.65\pm0.17}$ ****** | $-1.33\pm0.57$ | $2.37\pm0.97$ | $\boldsymbol{3.71\pm0.80}$ ****** | $\boldsymbol{0.21\pm0.09}$ ****** |
| Age completed education | $\boldsymbol{0.07\pm0.03}$ ****** | $-0.18\pm0.10$ | $\boldsymbol{-1.05\pm0.16}$ ****** | $\boldsymbol{-0.86\pm0.13}$ ****** | $\boldsymbol{0.07\pm0.01}$ ****** |
| Smoking: past/current smoker | $-0.004\pm0.415$ | $-0.77\pm0.38$ | $-0.29\pm0.64$ | $0.48\pm0.53$ | $0.05\pm0.06$ |
| Alcohol frequency | $-0.05\pm0.05$ | $-0.06\pm0.16$ | $0.06\pm0.27$ | $0.08\pm0.22$ | $\boldsymbol{-0.05\pm0.03}$ ****** |
| Hours of sleep | $0.05\pm0.06$ | $0.10\pm0.21$ | $0.34\pm0.37$ | $0.23\pm0.31$ | $-0.04\pm0.03$ |
| Total brain WM volume | $-0.002\pm0.002$ | $0.01\pm0.01$ | $0.002\pm0.012$ | $-0.01\pm0.01$ | $-0.001\pm0.001$ |
| Hippocampus volume | $0.17\pm0.10$ | $-0.21\pm0.34$ | $-0.55\pm0.57$ | $-0.34\pm0.47$ | $0.04\pm0.05$ |
| Caudate nucleus volume | $-0.22\pm0.11$ | $-0.33\pm0.35$ | $0.57\pm0.59$ | $0.90\pm0.49$ | $-0.10\pm0.06$ |
| Putamen volume | $0.16\pm0.09$ | $-0.50\pm0.31$ | $-0.85\pm0.53$ | $-0.35\pm0.44$ | $-0.02\pm0.05$ |
| Pallidum volume | $0.11\pm0.19$ | $-0.63\pm0.62$ | $0.58\pm1.05$ | $1.21\pm0.87$ | $0.10\pm0.10$ |
| Amygdala volume | $\boldsymbol{-0.46\pm0.17}$ ****** | $0.46\pm0.57$ | $1.82\pm0.96$ | $1.36\pm0.80$ | $-0.17\pm0.09$ |
| Nucleus accumbens volume | $-0.004\pm0.415$ | $0.51\pm1.37$ | $-0.19\pm2.32$ | $-0.70\pm1.92$ | $0.20\pm0.22$ |
| Prefrontal cortex GM volume | $0.02\pm0.01$ | $-0.09\pm0.05$ | $\boldsymbol{-0.24\pm0.08}$ ****** | $-0.16\pm0.07$ | $\boldsymbol{0.03\pm0.01}$ ****** |

**Supplementary Table 1.** Summary of the multivariable linear model that measures the association between thalamus volume with each independent cognitive test scores, as well as the Trail B time – Trail A time and maximum number of digits remembered correctly. Associations with significant FDR-corrected P-values were marked bold with (**) sign. DSST: Digit-symbol substitution test, TMT: Trail Making Test, NMT: Numeric Memory Test, GM: gray matter, WM: white matter.


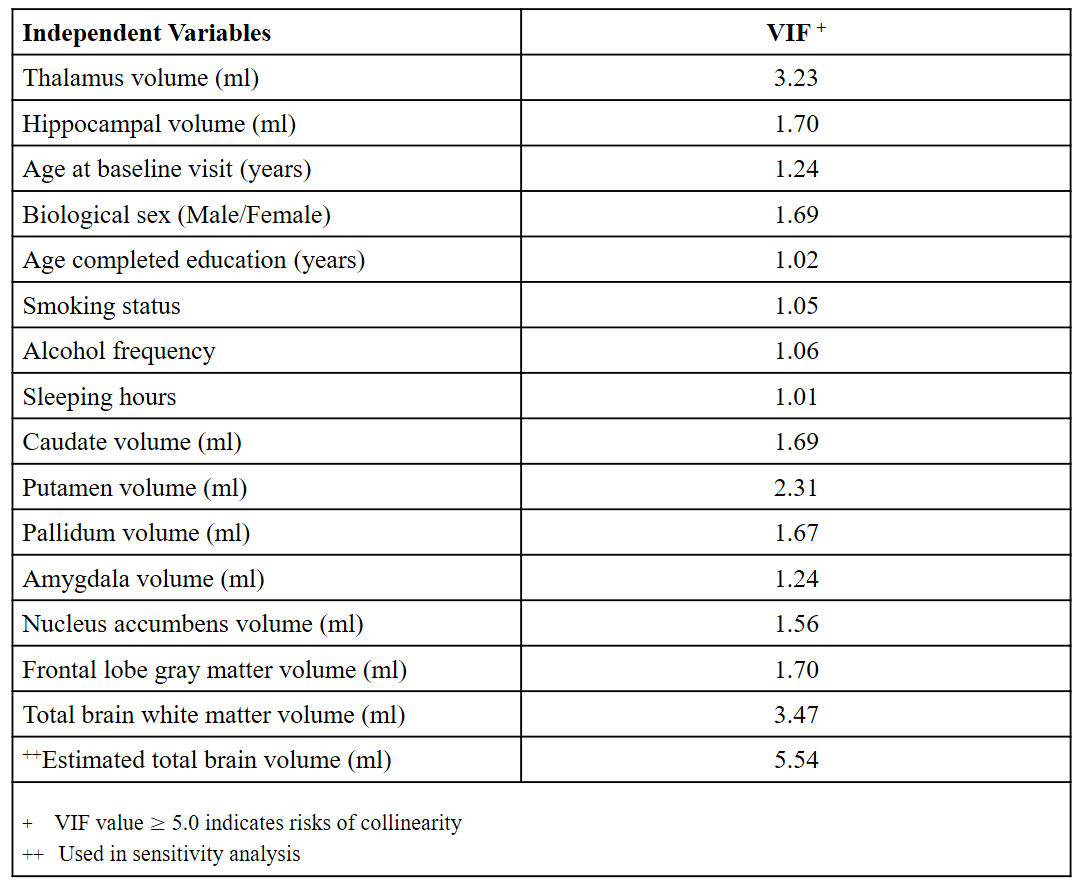


**Supplementary Table 2.** Summary of VIF values in the Aim-1 linear models and sensitivity analyses. VIF: variation inflation factor.

| **Demographic variables** | **Aim-1 Sample (**$\boldsymbol{\mu\pm\sigma}$**)** | **Aim-2 Sample (**$\boldsymbol{\mu\pm\sigma}$**)** | **T /** $\boldsymbol{\chi}^{\boldsymbol{2}}$ |
| --- | --- | --- | --- |
| **Age**  *(Years)* | $67.9\pm4.8$ | $66.9\pm4.7$ | **5.2 **** |
| **Sex** | Men: 1,991 (45.4%)  Women: 2,357 (54.6%) | Men: 335 (51.3%)  Women: 318 (48.7%) | **6.7 **** |
| **Age education ended**  *(Years)* | $17.2\pm2.3$ | $16.9\pm2.6$ | 1.5 |
| **Smoking Frequency**  *0=Never,*  *1= Past,*  *2= Current* | $0.51\pm0.64$ | $0.43\pm0.62$ | **2.8 **** |
| **Alcohol Frequency**  *1 = Daily*  *2 = 3-4 times a week*  *3 = 1-2 times a week*  *4 = 1-3 times a month*  *5 = Special occasions only*  *6 = Never* | $2.73\pm1.42$ | $2.65\pm1.38$ | 1.36 |
| **Diabetes**  *(Y/N)* | No: 4217  Yes: 127  n/a.: 4 | No: 637  Yes: 15  n/a. 1 | 0.59 |
| **Brain volume (GM+WM)**  *(L)* | $1.14\pm0.11$ | $1.15\pm0.11$ | **3.27 **** |
| **Thalamus volume**  *(ml)* | $14.95\pm1.34$ | $15.09\pm1.40$ | **2.42 **** |
| **Hippocampus volume**  *(ml)* | $7.52\pm0.85$ | $7.62\pm0.89$ | **2.89**** |
|  | | | |

**Supplementary Table 3.** Attrition table showing differences between participants who have both baseline and follow-up MRI scans (Aim-2) and those who were only available at baseline (Aim-1 only). T-value and chi-square values showing significant differences between Aim-1 and Aim-2 samples were marked bold with ** signs.

| **Model 4A:**  **Outcome variable:** Hippocampus volume at follow-up scan | | |
| --- | --- | --- |
| **Predictors** | $\boldsymbol{\beta\pm SE}$ | **P-value** |
| Mean fornix FA | $\boldsymbol{0.62\pm0.17}$ | **< 0.001 **** |
| **Confounders** | | |
| Baseline Hipp volume (ml) | $\boldsymbol{0.85\pm0.02}$ | **< 0.001 **** |
| Baseline Thal volume (ml) | $\boldsymbol{0.03\pm0.01}$ | **0.005 **** |
| Age at baseline visit (years) | $\boldsymbol{-0.01\pm0.002}$ | **< 0.001 **** |
| Sex (Men) | $0.03\pm0.03$ | 0.31 |
| Smoking frequency (0-2) | $0.02\pm0.01$ | **0.02 **** |
| Alcohol frequency (1-6) | $0.003\pm0.019$ | 0.86 |
| Time between scans (Years) | $-0.01\pm0.03$ | 0.59 |

| **Model 4B:**  **Outcome variable:** Thalamus volume at follow-up scan | | |
| --- | --- | --- |
| **Predictors** | $\boldsymbol{\beta\pm SE}$ | **P-value** |
| Mean fornix FA | $\boldsymbol{0.48\pm0.16}$ | **0.002 **** |
| **Confounders** | | |
| Baseline Hipp volume (ml) | $\boldsymbol{-0.04\pm0.02}$ | **0.007 **** |
| Baseline Thal volume (ml) | $\boldsymbol{0.98\pm0.01}$ | **< 0.001 **** |
| Age at baseline visit (years) | $-0.002\pm0.003$ | 0.44 |
| Sex (Men) | $0.02\pm0.03$ | 0.33 |
| Smoking frequency (0-2) | $0.003\pm0.008$ | 0.71 |
| Alcohol frequency (1-6) | $-0.009\pm0.018$ | 0.62 |
| Time between scans (Years) | $-0.014\pm0.025$ | 0.58 |

**Supplementary Table 4.** Summary of the linear model analysis of structural connectivity among mean fornix FA, hippocampal volume and thalamus volume. FA: Fractional anisotropy.

| **Model 5A:**  **Outcome variable:** Hippocampus volume at follow-up scan | | |
| --- | --- | --- |
| **Predictors** | $\boldsymbol{\beta\pm SE}$ | **P-value** |
| Baseline Thal volume (ml) | $\boldsymbol{0.04\pm0.01}$ | **0.002 **** |
| **Confounders** | | |
| Baseline Hipp volume (ml) | $\boldsymbol{0.87\pm0.02}$ | **< 0.001 **** |
| Age at baseline visit (years) | $\boldsymbol{-0.01\pm0.003}$ | **< 0.001 **** |
| Sex (Men) | $0.01\pm0.03$ | 0.82 |
| Smoking frequency (0-2) | $\boldsymbol{0.02\pm0.01}$ | **0.02 **** |
| Alcohol frequency (1-6) | $0.003\pm0.020$ | 0.89 |
| Time between scans (Years) | $-0.02\pm0.03$ | 0.57 |

| **Model 5B:**  **Outcome variable:** Thalamus volume at follow-up scan | | |
| --- | --- | --- |
| **Predictors** | $\boldsymbol{\beta\pm SE}$ | **P-value** |
| Baseline Hipp volume (ml) | $\boldsymbol{-0.03\pm0.02}$ | **0.04 **** |
| **Confounders** | | |
| Baseline Thal volume (ml) | $\boldsymbol{0.98\pm0.01}$ | **< 0.001 **** |
| Age at baseline visit (years) | $-0.01\pm0.003$ | 0.07 |
| Sex (Men) | $0.01\pm0.03$ | 0.78 |
| Smoking frequency (0-2) | $0.003\pm0.008$ | 0.74 |
| Alcohol frequency (1-6) | $-0.01\pm0.02$ | 0.57 |
| Time between scans (Years) | $-0.01\pm0.02$ | 0.57 |

**Supplementary Table 5.** Sensitivity analysis using linear regression models to validate the directionality of thalamus–hippocampus associations observed in the BLCS models. Thal: Thalamus, Hipp: Hippocampus, SE: Standard errors.

**Author Contribution Statements**

G.J. contributed to conceptualization, formal analyses, methodology, visualization, software, and writing-original draft. F.L, J.S.R. J.J.C. and W.S. were involved in conceptualization, writing-review and editing. H.C.M. contributed to methodology, software, and writing-review and editing. B.J.M. contributed to funding acquisition, project administration, conceptualization, methodology, supervision, and writing—review and editing.
